# Therapeutic effects of percutaneous endoscopic gastrostomy on survival in patients with amyotrophic lateral sclerosis: A meta-analysis

**DOI:** 10.1371/journal.pone.0192243

**Published:** 2018-02-06

**Authors:** Fang Cui, Liuqing Sun, Jianmei Xiong, Jianyong Li, Yangang Zhao, Xusheng Huang

**Affiliations:** 1 Department of Neurology, Hainan Branch of Chinese PLA General Hospital, Sanya, Hainan, China; 2 Department of Neurology, Chinese PLA General Hospital, Beijing, China; University Hospital Llandough, UNITED KINGDOM

## Abstract

Percutaneous endoscopic gastrostomy (PEG) is a method widely used for patients with amyotrophic lateral sclerosis (ALS); nevertheless, its effect on survival remains unclear. The purpose of this meta-analysis study was to determine the effects of PEG on survival in ALS patients. Relevant studies were retrieved from PubMed, EmBase, and the Cochrane Library databases, from inception to June 2017. Studies comparing PEG with other procedures in ALS patients were included. Odds ratios (ORs) in a random-effects model were used to assess the survival at different follow-up periods. Briefly, ten studies involving a total of 996 ALS patients were included. Summary ORs indicated that PEG administration was not associated with 30-day (OR = 1.59; 95%CI 0.93–2.71; P = 0.092), 10-month (OR = 1.25; 95%CI 0.72–2.17; P = 0.436), and 30-month (OR = 1.28; 95% CI 0.77–2.11; P = 0.338) survival rates, while they showed a beneficial effect in 20-month survival rate (OR = 1.97; 95%CI 1.21–3.21; P = 0.007). The survival rate was significantly prominent in reports published before 2005, and in studies with a retrospective design, sample size <100, mean age <60.0 years, and percentage male ≥50.0%. To sum up, these findings suggested that ALS patients administered with PEG had an increased 20-month survival rates, while there was no significant effect in 30-day, 10-month, and 30-month survival rates.

## Introduction

Amyotrophic lateral sclerosis (ALS) is a progressive degeneration of motor neurons, involving the upper and lower motor neurons of the spinal cord, brainstem, and cerebral cortex, that causes disability and death within three to five years following diagnosis [1–3]. ALS is cartherized by stiff muscles and muscle twitching, which in the majority of cases causes a progressive weakness and wasting of muscles which control the movement, breathing, and swallowing [4]. Malnutrition has been associated with a linear decline in muscle strength which increases the risk of death in patients with ALS [5–7]. Specifically, ALS patients with severe malnutrition suffer from muscle atrophy, muscle weakness, and diaphragmatic paralysis [8, 9]. Therefore, effective nutritional care is particularly important in ALS patients.

Currently, gastrostomy that has shown to improve the disease prognosis, is widely used in ALS patients [10, 11]. Two commonly used approaches for percutaneous gastrostomy are radiologic and endoscopic methods [12]. According to the American Academy of Neurology, ALS Practice Parameter guidelines, ALS patients with symptomatic dysphagia and vital capacity exceeding 50% of the predicted value should undergo percutaneous endoscopic gastrostomy (PEG). Nevertheless, only 13% of patients are administered with PEG due to lack of sufficient evidence for the effectiveness of PEG in ALS patients [13]. A previous meta-analysis demonstrated undefined effects of PEG on survival due to limited evidence, while PEG tubes have been associated with prolonged life in older individuals [14]. However, there was limited evidence demonstrating that PEG prolongs the survival in ALS patients [4]. Although numerous studies have evaluated the therapeutic effects of PEG on survival in ALS patients, supportive high-quality clinical evidence is not available. Hence, a more robust analysis of all the available studies is highly necessary. Here, we attempted a large-scale examination of the available studies comparing PEG and other procedures with regard to the survival in ALS patients.

## Materials and methods

### Data sources, search strategy, and selection criteria

This review was performed according to the Preferred Reporting Items for Systematic Reviews and Meta-Analysis Statement guidelines issued in 2009 (S1 Checklist) [15]. The PubMed, EmBase, and Cochrane library were searched for articles assessing the effectiveness of PEG in ALS patients from the inception date to June 2017. The keywords used were the following: “(percutaneous endoscopic gastrostomy or PEG)” and “amyotrophic lateral sclerosis” or “sclerosis, amyotrophic lateral” or “Charcot disease” or “motor neuron disease”. The detailed search strategy in PubMed was presented in **S1 File**. The search was limited to the articles published in English language. The abstracts and virtual meeting presentations were searched to identify for relevant unpublished studies. We also hand-searched the journals for published relevant data, as well as the reference lists of all the retrieved articles and relevant review articles.

The literature search was independently undertaken by two investigators using a standardized approach, while any inconsistencies were settled by the primary author. The inclusion criteria were: (1) study with prospective or retrospective design; (2) all included patients had ALS; (3) patients administered either with PEG or non-PEG interventions; (4) availability of survival rate in each group. Studies were excluded if: (1) patients were diagnosed with other diseases; (2) the study with inappropriate control; and (3) studies without adequate data.

### Data collection and quality assessment

Data extraction and assessment were conducted independently by two investigators. Publication data (first author’s name and publication year), study characteristics (country, study design, sample size, mean age, percentage male, intervention, and control), and survival rates at different follow-up durations were extracted. Disagreements were resolved by a third reviewer or consensus-based discussion. 30-day survival rate was the primary endpoint in the present meta-analysis study, while the secondary endpoints included 10-month, 20-month, and 30-month survival rates.

The Newcastle-Ottawa Scale (NOS), which is quite comprehensive and has been partially validated for evaluating the quality of observational studies in the meta-analysis, was used to assess the methodological quality [16]. NOS were based on the following 3 subscales: selection (4 items), comparability (1 item), and outcome (3 items). A “star system” (range, 0–9) was developed for assessment. The quality assessment was performed independently by two investigators. The data were examined and adjudicated independently by an additional investigator based on original reports.

### Statistical analysis

The therapeutic effects of PEG on survival were assessed based on the number of surviving patients and sample size in each group for every study. Odds ratios (ORs) with corresponding 95% confidence intervals (CIs) were employed as summary statistics with random-effects model. The results from the random-effects model imply that the true underlying effect varies across the included studies [17, 18]. Study heterogeneity was analyzed by Q statistics, with P<0.10 denoting statistical significance. The degree of heterogeneity was based on the I^2^ statistics, with I^2^>50% indicating significant heterogeneity and data with I^2^>75% were not included in the final analysis [19, 20]. Sensitivity analysis was carried out by removing each individual study from the overall analysis to evaluate its influence on the obtained results [21]. Subgroup analyses were also conducted based on publication year, study design, sample size, mean age, and percentage of males. Furthermore, Chi-square test and meta-regression were used to assess various subgroups [22]. The Egger’s [23] and Begg’s [24] tests were used to statistically assess publication bias for major cardiovascular outcomes. Two-sided P<0.05 was considered statistically significant. Statistical analyses were performed with the STATA software (version 12.0; Stata Corporation, College Station, TX, USA).

## Results

**Fig 1:**
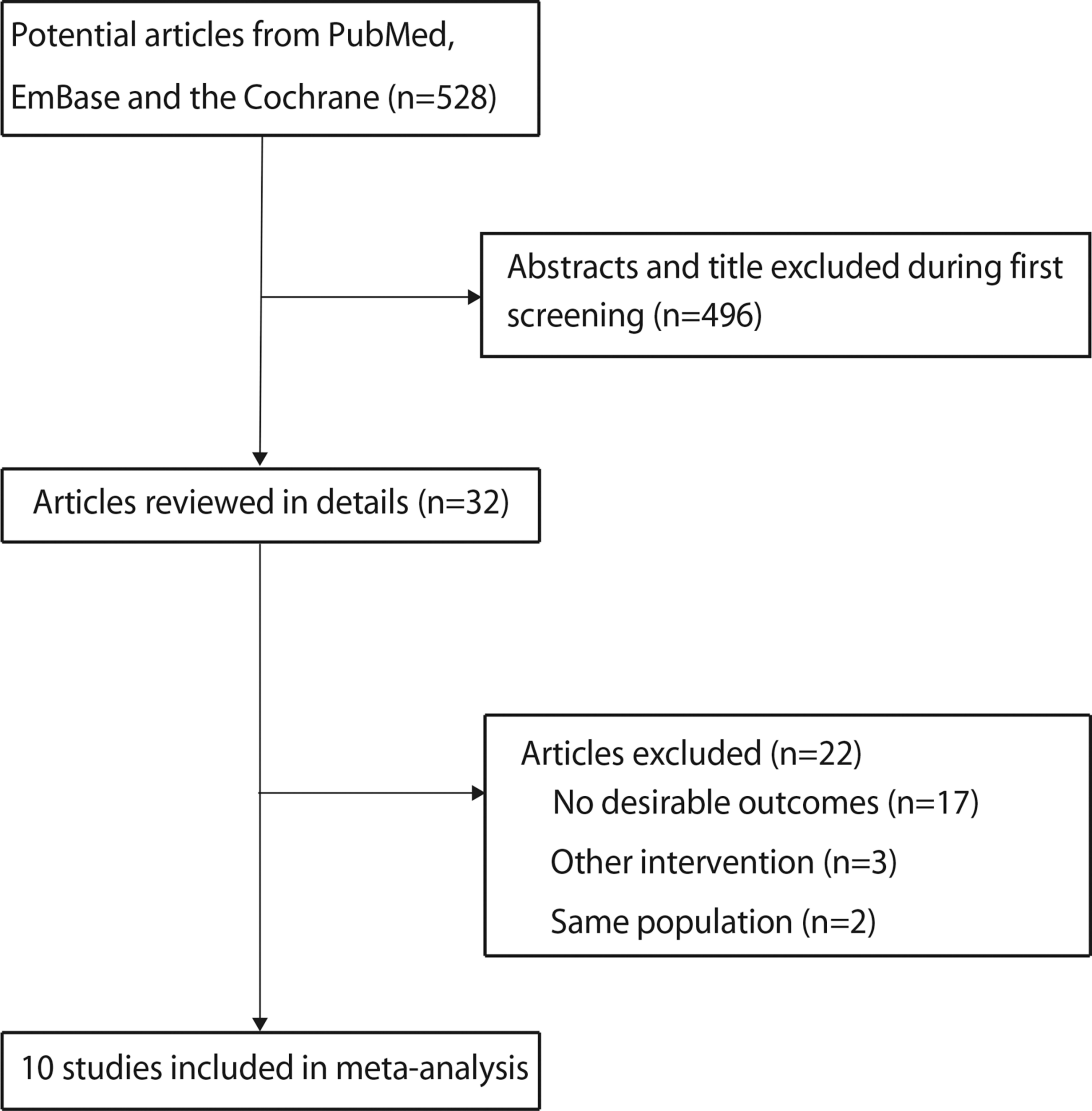
Flow diagram of the literature search and selection process.

The study-selection process is shown in **Fig 1**. A total of 528 articles (187 from PubMed, 326 from EmBase, and 15 from Cochrane library) were retrieved during the initial electronic search, while 496 articles were irrelevant or duplicate, and thus were excluded. Meanwhile, 32 potentially eligible studies were selected. After detailed evaluation, 10 studies were selected for the final meta-analysis [10, 25–33]. Twenty-two studies were excluded due to the following reasons: survival rate data was not reported in 17 studies; three studies included patients with other interventions; and 2 studies reported sample patient population. A manual search of the reference lists of the latter studies yielded no further eligible studies. General characteristics of the included studies are presented in **Table 1**.

**Table 1:** Baseline characteristic of studies included in the systematic review and meta-analysis.

Study	Country	Study design	Sample size	Age	Percentage of males(%)	Intervention	Control	NOS score
Shaw 2006 [25]	UK	Retrospective	98	61.0	50.0	PEG	RIG or NG	7
Mathus-Vliegen 1994 [26]	The Netherlands	Prospective	68	57.0	48.5	PEG	Other	7
Mazzini 1995 [10]	Italy	Prospective	66	59.9	51.5	PEG	Other	7
Spataro 2011 [27]	Italy	Retrospective	150	60.5	55.3	PEG	Other	8
Blondet 2010 [28]	France	Retrospective	40	66.1	42.5	PEG	PRG	7
Thornton 2002 [29]	Ireland	Retrospective	36	54.0	52.8	PEG	PRG	7
Allen 2013 [30]	USA	Retrospective	108	60.6	59.3	PEG	RIG	8
Desport 2005 [31]	France	Prospective	50	65.9	52.0	PEG	RIG	7
ProGas Study Group 2015 [32]	UK	Prospective	330	64.4	55.0	PEG	RIG or PIG	8
Chio 2004 [33]	Italy	Retrospective	50	67.0	50.0	PEG	PRG	7

*PEG, percutaneous endoscopic gastrostomy; RIG, radiological inserted gastrostomy; NG, nasogastric tube; PRG, percutaneous radiologic gastrostomy; PIG, per-oral image-guided gastrostomy; NOS, Newcastle-Ottawa Scale

Among 10 included studies (including a total of 996 ALS patients), 4 [10, 25–33] were prospective studies and 6 were retrospective trials. The mean age of ALS patients was 54.0–67.0 years, and 36–330 patients were included in each study. Two studies were conducted in UK [25, 32], 3 in Italy [10, 27, 33], 2 in France [28, 31], 1 in the Netherlands [26], 1 in Ireland [29], and 1 in USA [30]. Study quality was evaluated using the NOS scale. Three studies had a score of 8 [27, 30, 32], while 7 had a score of 7 [10, 25, 26, 28, 29, 31, 33], respectively.

**Fig 2:**
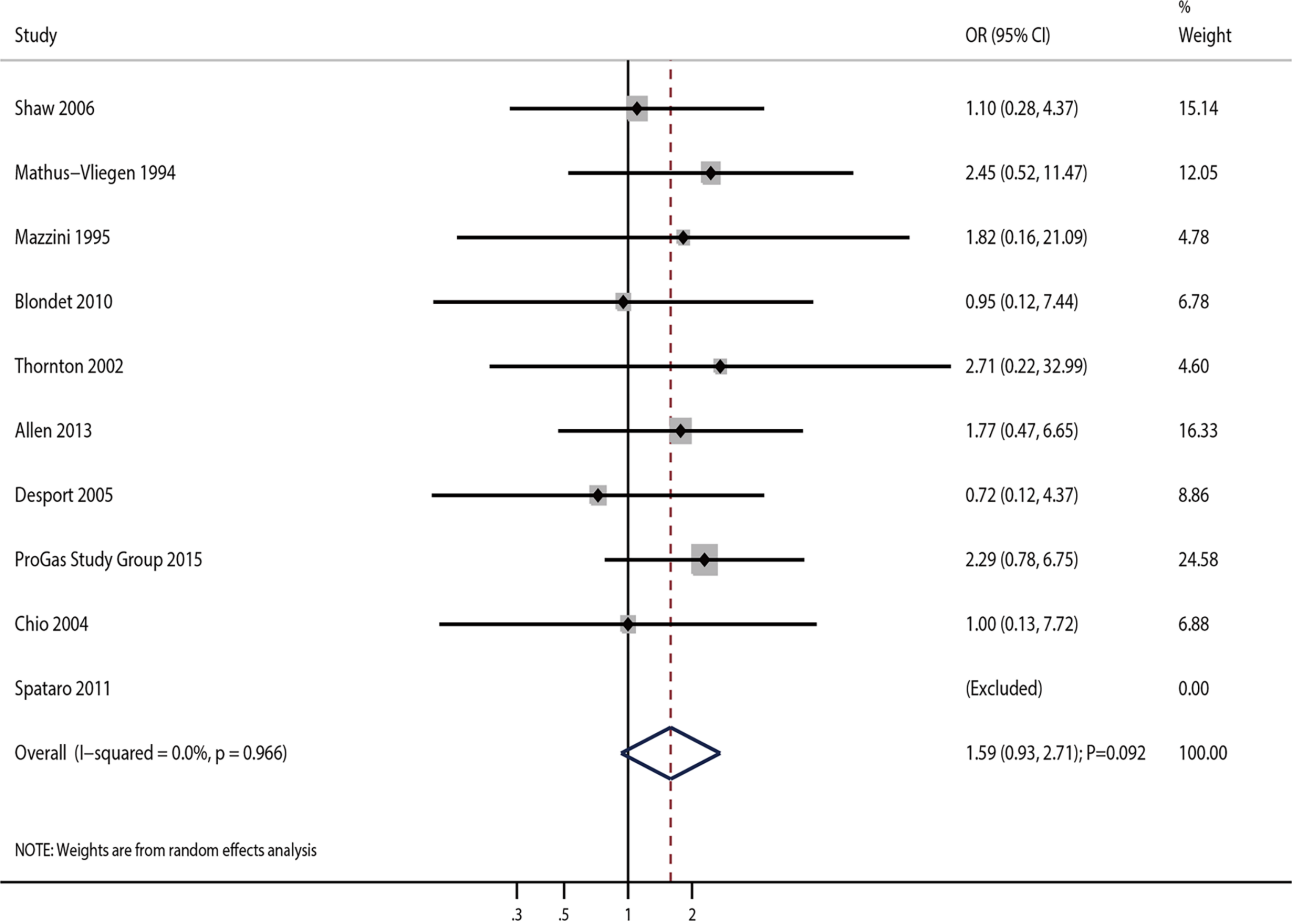
Association of PEG use with 30-day survival rate.

The effects of PEG on 30-day survival rate in ALS patients was reported in ten studies. The summary OR indicated that PEG administration in ALS patients was not associated with 30-day survival rate (OR = 1.59; 95%CI 0.93–2.71; P = 0.092; **Fig 2**), and with no overt heterogeneity (I^2^ = 0.0%; P = 0.966). Sensitivity analysis was performed, and sequential removal of each study from the pooled analysis did not significantly affect the overall results of the meta-analysis (**Table 2**). Furthermore, subgroup analysis data were consistent with the overall findings, and no pre-defined factors affected the therapeutic effects of PEG on 30-day survival rate (**Table 3**). In addition, sample size, mean age, and gender were not considered as significant factors on 30-day survival rate (**S1 Table**). Finally, there was no significant publication bias among the included studies (P value for the Egger’s test, 0.402; P value for the Begg’s test, 0.754; **Table 4**).

**Table 2:** Sensitivity analysis.

Outcomes	Excluding study	OR and 95% CI	P value	Heterogeneity (%)	P value for heterogeneity
**30-day survival rate**	Shaw 2006	1.69 (0.95–3.03)	0.076	0.0	0.955
	Mathus-Vliegen 1994	1.49 (0.84–2.65)	0.168	0.0	0.957
	Mazzini 1995	1.58 (0.91–2.73)	0.105	0.0	0.935
	Spataro 2011	1.59 (0.93–2.71)	0.092	0.0	0.966
	Blondet 2010	1.65 (0.95–2.87)	0.078	0.0	0.951
	Thornton 2002	1.55 (0.89–2.68)	0.120	0.0	0.947
	Allen 2013	1.55 (0.86–2.79)	0.141	0.0	0.937
	Desport 2005	1.71 (0.98–3.00)	0.060	0.0	0.979
	ProGas Study Group 2015	1.41 (0.76–2.61)	0.278	0.0	0.969
	Chio 2004	1.64 (0.94–2.86)	0.080	0.0	0.949
**10-month survival rate**	Shaw 2006	1.25 (0.65–2.39)	0.500	55.5	0.028
	Mathus-Vliegen 1994	1.28 (0.69–2.39)	0.434	54.9	0.030
	Mazzini 1995	1.17 (0.61–2.24)	0.641	54.7	0.031
	Spataro 2011	1.25 (0.69–2.25)	0.458	55.5	0.028
	Blondet 2010	1.12 (0.62–2.02)	0.709	50.0	0.051
	Thornton 2002	1.09 (0.64–1.87)	0.741	41.8	0.100
	Desport 2005	1.44 (0.81–2.53)	0.212	44.6	0.082
	ProGas Study Group 2015	1.18 (0.56–2.51)	0.660	56.2	0.025
	Chio 2004	1.38 (0.91–2.10)	0.125	20.2	0.269
**20-month survival rate**	Shaw 2006	1.94 (1.16–3.26)	0.012	0.0	0.490
	Mathus-Vliegen 1994	2.04 (1.22–3.40)	0.006	0.0	0.514
	Mazzini 1995	1.68 (0.99–2.85)	0.055	0.0	0.832
	Spataro 2011	2.13 (1.16–3.92)	0.015	0.0	0.510
	Blondet 2010	1.86 (1.11–3.12)	0.018	0.0	0.545
	Thornton 2002	2.02 (1.20–3.39)	0.008	0.0	0.497
	Desport 2005	2.18 (1.31–3.62)	0.003	0.0	0.762
**30-month survival rate**	Shaw 2006	1.25 (0.74–2.10)	0.409	0.0	0.928
	Mathus-Vliegen 1994	1.28 (0.76–2.17)	0.352	0.0	0.913
	Spataro 2011	1.34 (0.61–2.97)	0.463	0.0	0.917
	Blondet 2010	1.22 (0.73–2.03)	0.451	0.0	0.993
	Thornton 2002	1.32 (0.77–2.23)	0.310	0.0	0.929
	Desport 2005	1.30 (0.77–2.18)	0.320	0.0	0.923

*OR, odds ratio; CI, confidence interval

**Table 3:** Subgroup analysis.

Outcomes	Group	OR and 95%CI	P value	Heterogeneity (%)	P value for heterogeneity	P value for heterogeneity between subgroups
**30-day survival rate**	Publication year					
	2005 or after	1.48(0.78–2.78)	0.228	0.0	0.798	0.674
	Before 2005	1.91(0.70–5.22)	0.210	0.0	0.905	
	Study design					
	Prospective	1.86(0.87–3.96)	0.108	0.0	0.723	0.562
	Retrospective	1.35(0.63–2.89)	0.436	0.0	0.947	
	Sample size					
	100 or larger	2.06(0.89–4.77)	0.090	0.0	0.767	0.424
	< 100	1.32(0.66–2.65)	0.433	0.0	0.947	
	Mean age (years)					
	60 or older	1.43(0.78–2.61)	0.250	0.0	0.878	0.456
	< 60	2.34(0.74–7.46)	0.149	0.0	0.972	
	Percentage male (%)					
	50 or larger	1.55(0.86–2.81)	0.147	0.0	0.933	0.877
	<50	1.74(0.51–5.99)	0.378	0.0	0.469	
**10-month survival rate**	Publication year					
	2005 or after	1.22(0.77–1.93)	0.398	14.4	0.322	1.000
	Before 2005	1.06(0.25–4.54)	0.938	74.3	0.009	
	Study design					
	Prospective	1.17(0.77–1.79)	0.457	10.6	0.340	1.000
	Retrospective	1.28(0.34–4.81)	0.717	68.3	0.013	
	Sample size					
	100 or larger	1.32(0.85–2.05)	0.217	0.0	0.859	1.000
	< 100	1.18(0.53–2.65)	0.685	62.4	0.014	
	Mean age (years)					
	60 or older	0.96(0.45–2.05)	0.919	56.2	0.044	0.256
	< 60	1.91(0.80–4.59)	0.147	34.8	0.216	
	Percentage male (%)					
	50 or larger	1.13(0.58–2.22)	0.721	56.6	0.032	0.577
	<50	1.69(0.52–5.52)	0.386	38.5	0.202	
**20-month survival rate**	Publication year					
	2005 or after	1.74(0.95–3.18)	0.074	0.0	0.562	0.492
	Before 2005	2.49(1.09–5.68)	0.031	0.0	0.381	
	Study design					
	Prospective	1.81(0.53–6.18)	0.344	48.3	0.145	0.929
	Retrospective	1.94(1.07–3.50)	0.028	0.0	0.902	
	Sample size					
	100 or larger	1.71(0.76–3.87)	0.194	-	-	0.677
	< 100	2.13(1.16–3.92)	0.015	0.0	0.510	
	Mean age (years)					
	60 or older	1.74(0.95–3.18)	0.074	0.0	0.562	0.492
	< 60	2.49(1.09–5.68)	0.031	0.0	0.381	
	Percentage male (%)					
	50 or larger	1.93(1.12–3.31)	0.018	0.0	0.422	0.859
	<50	2.16(0.71–6.58)	0.177	0.0	0.465	
**30-month survival rate**	Publication year					
	2005 or after	1.33(0.76–2.32)	0.321	0.0	0.835	0.765
	Before 2005	1.10(0.36–3.36)	0.872	0.0	0.861	
	Study design					
	Prospective	1.12(0.32–3.89)	0.858	0.0	0.875	0.822
	Retrospective	1.31(0.76–2.26)	0.333	0.0	0.824	
	Sample size					
	100 or larger	1.23(0.65–2.35)	0.523	-	-	0.870
	< 100	1.34(0.61–2.97)	0.463	0.0	0.917	
	Mean age (years)					
	60 or older	1.33(0.76–2.32)	0.321	0.0	0.835	0.765
	< 60	1.10(0.36–3.36)	0.872	0.0	0.861	
	Percentage male (%)					
	50 or larger	1.22(0.71–2.08)	0.474	0.0	0.971	0.635
	<50	1.73(0.45–6.70)	0.426	0.0	0.473	

*OR, odds ratio; CI, confidence interval

**Table 4:** Publication bias analysis.

Outcomes	P value for the Egger’s test	P value for the Begg’s test
**30-day survival rate**	0.402	0.754
**10-month survival rate**	0.546	0.754
**20-month survival rate**	0.816	0.368
**30-month survival rate**	0.460	0.260

**Fig 3:**
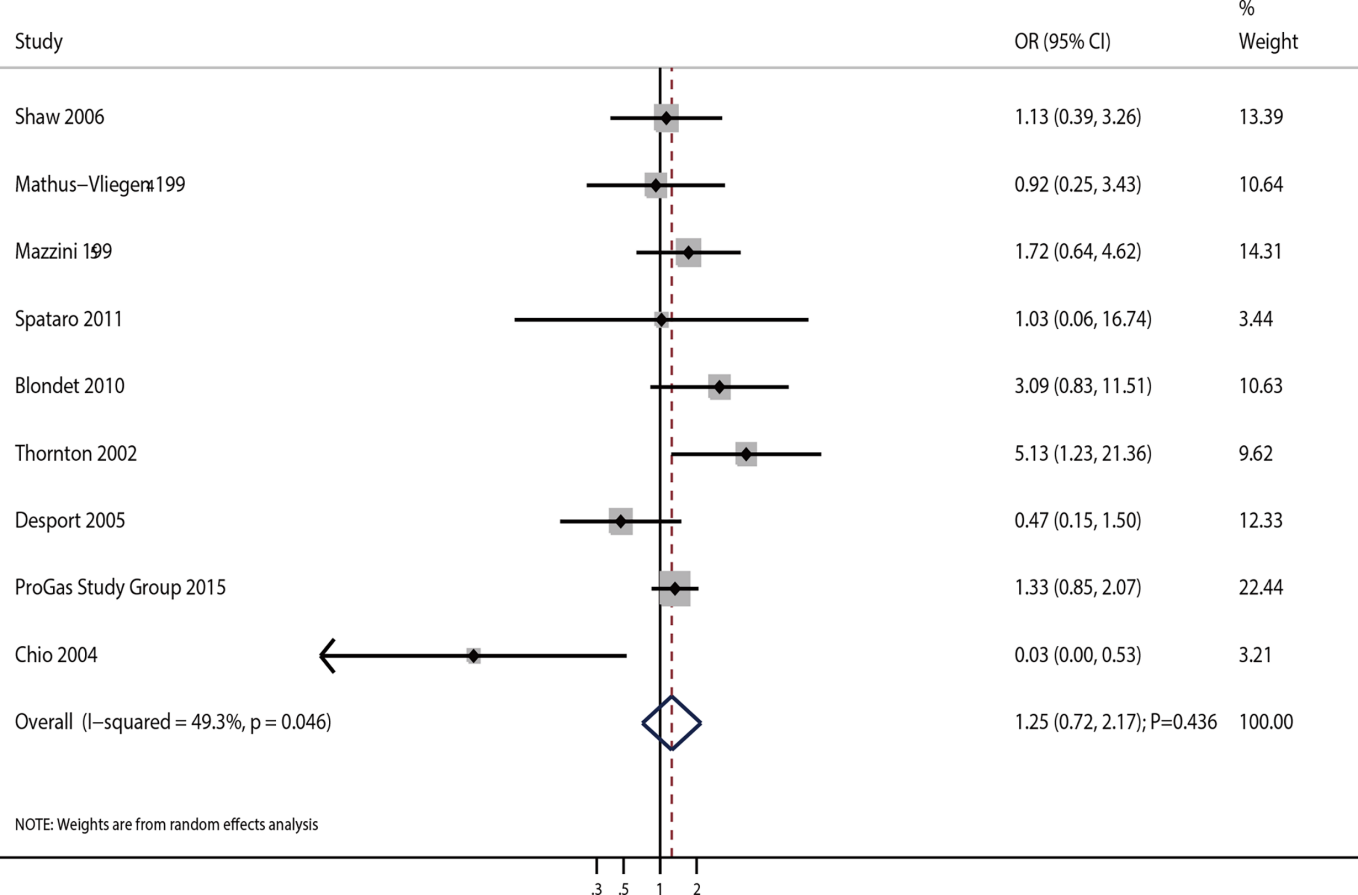
Association of PEG use with 10-month survival rate.

A total of 9 studies evaluated the effects of PEG on 10-month survival rate in ALS patients. Our results showed no significant association between PEG and 10-month survival rate (OR = 1.25; 95%CI 0.72–2.17; P = 0.436; Fig 3). Although substantial heterogeneity was observed in the magnitude of effects across studies (I^2^ = 49.3%; P = 0.046), sequential exclusion of each study from the pooled data did not affect the overall results (**Table 2**). Furthermore, meta-regression analyses suggested that sample size, mean age, and gender (percentage of male patients) did not affect the 10-month survival rate (**S1 Table**). Findings of subgroup analysis were consistent with the overall analysis (**Table 3**) and no publication bias was observed (P value for the Egger’s test, 0.546; P value for the Begg’s test, 0.754; **Table 4**).

**Fig 4:**
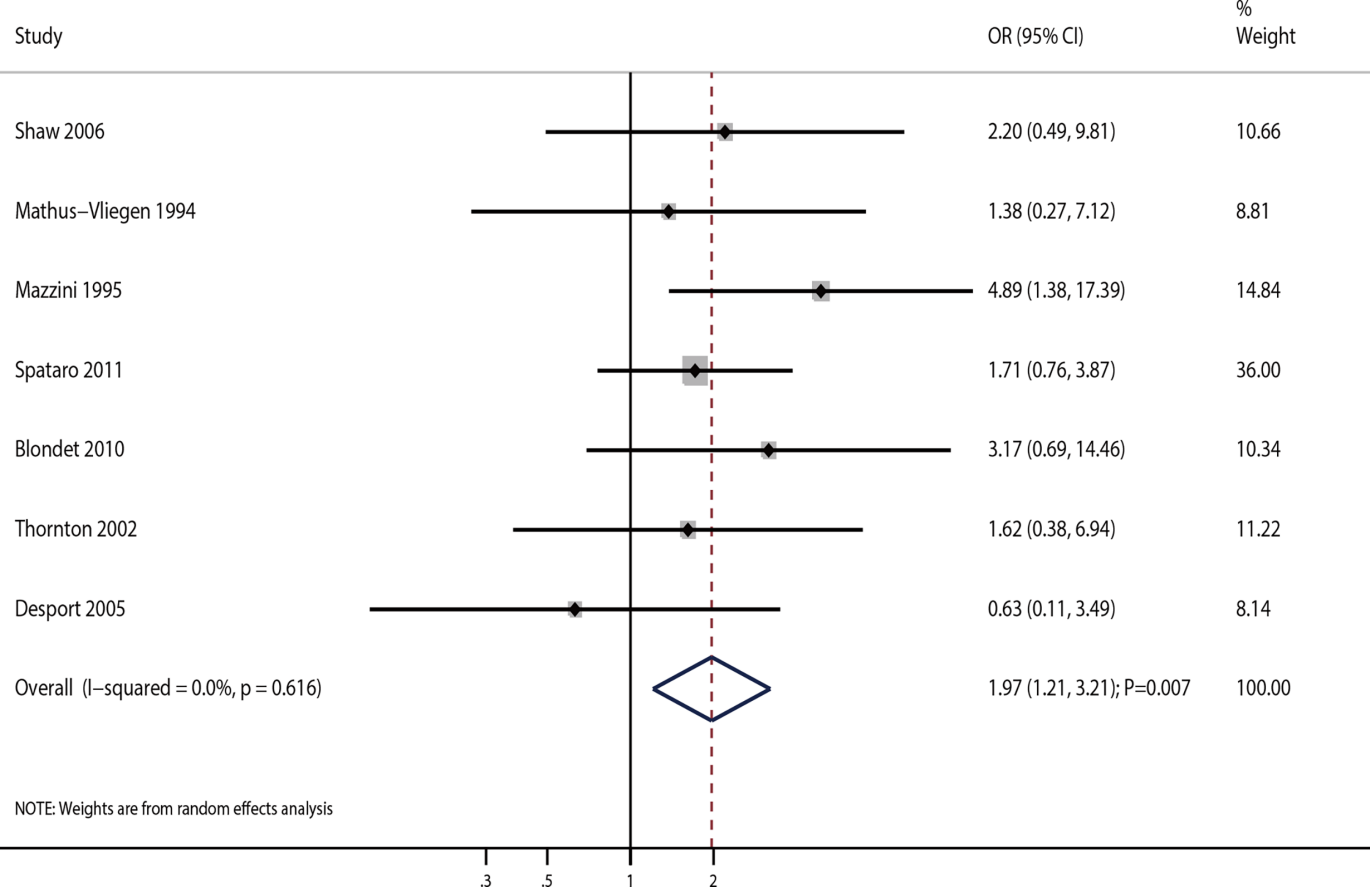
Association of PEG use and 20-month survival rate.

A total of 7 studies assessed the effects of PEG on 20-month survival rate in ALS patients. Pooled analysis of 20-month survival rate indicated a beneficial effect of PEG compared with other procedures (OR = 1.97; 95%CI 1.21–3.21; P = 0.007; **Fig 4**). There was no significant heterogeneity among the included studies (I^2^ = 0.0%; P = 0.616). Sequential exclusion of each study from the pooled data did not affect the overall conclusion (**Table 2**). The results of meta-regression analyses found that sample size, mean age, and gender (percentage of male patients) were not correlated with 20-month survival rate (**S1 Table**). Subgroup analysis suggested significant improvement in 20-month survival rate, which was prominent in studies published before 2005, and those with a retrospective design, sample size <100, mean age <60.0 years, and percentage of male patients ≥50.0% (Table 3). Egger’s (P = 0.816) and Begg’s (P = 0.368) tests suggested no significant publication bias for 20-month survival rate (**Table 4**).

**Fig 5:**
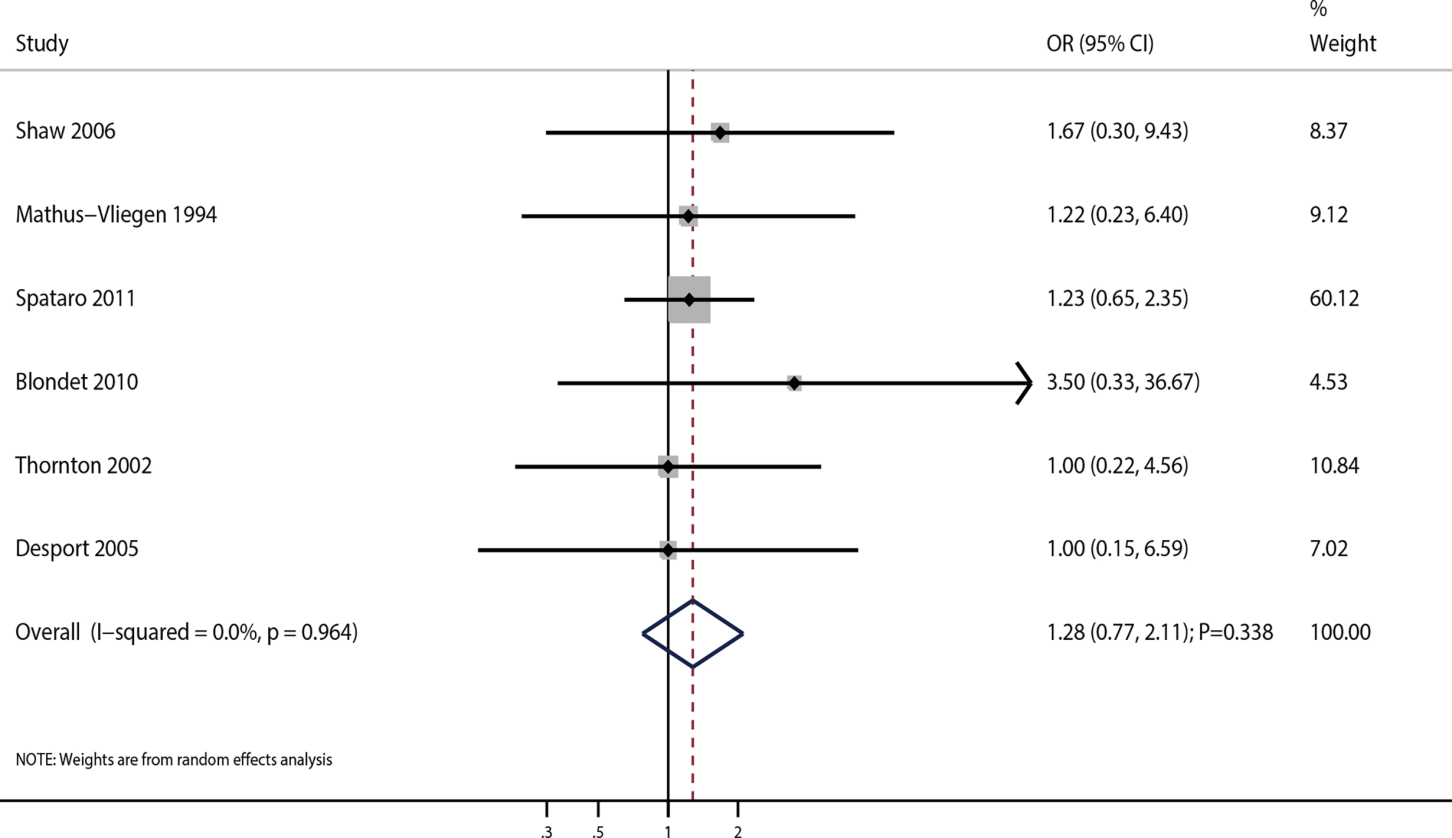
Association of PEG use and 30-month survival rate.

A total of 6 studies reported the effects of PEG on 30-month survival rate in ALS patients. Pooled analysis indicated that there was no association between PEG and 30-month survival rate (OR = 1.28; 95%CI 0.77–2.11; P = 0.338; **Fig 5**). Although no heterogeneity was detected across the included studies (I^2^ = 0.0%; P = 0.964), sensitivity analysis was conducted. After sequential exclusion of each study from the pooled analysis, the conclusion was not affected (**Table 2**). Sample size, mean age, and gender (percentage of male patients) did not significantly affect the 30-month survival rate (**S1 Table**). There were no significant differences in 30-month survival rate based on subgroup analysis (**Table 3**). No significant publication bias was observed among the included studies (P value for the Egger’s test was 0.460; P value for the Begg’s test was 0.260; **Table 4**).

## Discussion

The current meta-analysis explored the therapeutic effects of PEG and survival rates at different follow-up durations based on a total of 996 ALS patients with a broad range of ethnicities assessed in 4 prospective and 6 retrospective studies. The obtained results suggested that compared to control, PEG had no effect on 30-day, 10-month, and 30-month survival rates. Yet, PEG showed a significant increase in 20-month survival rates. Sensitivity and subgroup analysis findings were consistent with the overall analysis for 30-day, 10-month, and 30-month survival rates. The therapeutic effects of PEG on 20-month survival rate were outstanding in studies published before 2005, and in studies with a retrospective design, sample size <100, mean age <60.0 years, and percentage of male patients ≥ 50.0%.

Malnutrition is an independent prognostic factor for muscle weakness and death in patients with ALS. Both dysphagia and hypermetabolism are significant risk factors for malnutrition, and they remain to be a major problem in ALS patients [34–36]. Currently, enteral nutrition is widely used to treat dysphagia in ALS patients. Therefore, ALS patients with dysphagia undergo PEG as a routine procedure for nutrition improvement, although the effects of PEG on survival remain undefined. Shaw et al [25] have reported equivalent post-procedure survival rates between PEG and radiological inserted gastrostomy, which have been found to decrease the morbidity and mortality. Finally, poor survival rate was expectedly observed in the nasogastric tube group, even though most of the patients who participated in the study were in pre-terminal stages of ALS. Mathus-Vliegen et al [26] have suggested PEG is a successful and safe procedure in highly disabled ALS patients with respiratory compromise and far-advanced neurologic disease. Mazzini et al [10] have indicated that long-term PEG use is associated with significantly improved survival rate in ALS patients with bulbar involvement. The possible reason for this is that most ALS patients die at home due to broncho-pneumonic complications, while PEG is associated with low incidence of pneumonia and cachexia. Spataro et al [27] have retrospectively analyzed 150 ALS patients with dysphagia, and found that PEG significantly improved survival, with fewer side effects. Furthermore, another study has also suggested that patients with spinal-onset benefitted more compared to their bulbar-onset counterparts. Blondet et al [28] have suggested that percutaneous radiologic gastrostomy is more efficacious and well-tolerated compared to PEG in ALS patients, likely because PEG has been associated with an increased risk of respiratory decompensation in ALS patients. Thornton et al [29] have demonstrated that percutaneous radiologic gastrostomy is associated with increased success rate compared with PEG. Allen et al [30] have indicated that PEG significantly increased the risk of post-procedure aspiration, while no significant difference has been observed in 30-day survival rate between the PEG and radiologic gastrostomy groups. Desport et al [31] have found no significant difference between PEG and RIG in survival rates, revealing a higher failure rate in the PEG group compared to RIG group. A study by ProGas Study Group indicated no significant difference in the survival rate and adverse events among PEG, radiologically inserted gastrostomy, and per-oral image-guided gastrostomy [32]. Chio et al have demonstrated that percutaneous radiological gastrostomy is superior to PEG in terms of survival in ALS patients with moderate or severe respiratory impairment [33]. They also proposed the use of nasogastric tube in the percutaneous radiological gastrostomy procedures, which is considered more favorable in patients with masseter spasm as they cannot open the mouth. The important strengths of the present study involve comprehensive inclusion of relevant studies with larger statistical power compared with any individual study. In addition, this report was based on 10 studies that were conducted in different countries, and that have broad characteristics, suggesting the broad applicability of the present findings.

The present meta-analysis suggested that ALS patients receiving PEG were associated with higher incidence of 20-month survival, while no significant differences were observed for 30-day, 10-month, and 30-month survival rates. However, due to chance these results might not be enough to substantiate a causal relationship between the impact of PEG on survival rate at different follow-up durations. The possible reason for this could be that the pooled results were obtained from various included studies. During the planning stages, the treatment effect of PEG on survival rate should be conducted according to nutritional status, while most of the included studies did not provide the information regarding the nutritional status and other baseline characteristics of ALS patients. Furthermore, ALS patients can have hypermetabolism, thus the caloric requirements of patients can contribute to the lower energy intake than energy expenditure [37].

Although subgroup analysis suggested prominent therapeutic effects of PEG in studies published before 2005, and in studies with a retrospective design, sample size <100, mean age <60.0 years, and percentage of male patients ≥50.0% for 20-month survival rate in ALS patients, no significant difference between various subgroups was observed. The potential reason for the studies conducted before 2005 was associated with different nutritional status. In addition, our study patients from different countries were correlated with nutritional status, while all the included studies were conducted in highly developed countries. Additionally, the study design and sample size were correlated with evidence level and statistical power. Finally, these factors might disprove the significant impact on 20-month survival in ALS patients administered with PEG. Also, the number of studies included in these subsets was very small, with lower event rates than expected, yielding broad confidence intervals, i.e. no statistically significant differences.

The present study has few limitations which are as follows: (1) several important factors, including general characteristics and clinical conditions affecting the survival rates at different follow-up durations, were not adjusted; (2) based on the published studies, publication bias was an inevitable problem in this analysis; (3) pooled data were used for analysis (individual data were not available), which restricted us from performing a substantially detailed analyses with more comprehensive results.

In conclusion, the current findings suggest that PEG significantly increases 20-month survival rates in ALS patients, with no significant effect on 30-day, 10-month, and 30-month survival rates. These results are consistent, and PEG should be recommended for ALS patients. Future large scale randomized controlled trials are required to confirm the therapeutic effects of PEG on survival rates in ALS patients.

## Supporting information

S1 Checklist PRISMA checklist. (DOC)

S1 File The detailed search strategy in PubMed. (DOC)

S1 Table Meta-regression for investigated outcomes according to sample size, mean age, and percentage male. (DOC)

## References

[1] Lee JR, Annegers JF, Appel SH. Prognosis of amyotrophic lateral sclerosis and the effect of referral selection. J Neurol Sci. 1995;132(2):207–15. 8543950 10.1016/0022-510x(95)00154-t

[2] Preux PM, Couratier P, Boutros-Toni F, Salle JY, Tabaraud F, Bernet-Bernady P, et al Survival prediction in sporadic amyotrophic lateral sclerosis. Age and clinical form at onset are independent risk factors. Neuroepidemiology. 1996;15(3):153–60. 8700307 10.1159/000109902

[3] Traynor BJ, Codd MB, Corr B, Forde C, Frost E, Hardiman OM. Clinical features of amyotrophic lateral sclerosis according to the El Escorial and Airlie House diagnostic criteria: A population-based study. Arch Neurol. 2000;57(8):1171–6. 10927797 10.1001/archneur.57.8.1171

[4] McDermott CJ, Shaw PJ. Diagnosis and management of motor neurone disease. BMJ. 2008;336(7645):658–62. doi: 10.1136/bmj.39493.511759.BE 18356234 10.1136/bmj.39493.511759.BE PMC2270983

[5] Desport JC, Preux PM, Truong TC, Vallat JM, Sautereau D, Couratier P. Nutritional status is a prognostic factor for survival in ALS patients. Neurology. 1999;53(5):1059–63. 10496266 10.1212/wnl.53.5.1059

[6] Christensen PB, Hojer-Pedersen E, Jensen NB. Survival of patients with amyotrophic lateral sclerosis in 2 Danish counties. Neurology. 1990;40(4):600–4. 2320232 10.1212/wnl.40.4.600

[7] Norris F, Shepherd R, Denys E, U K, Mukai E, Elias L, et al Onset, natural history and outcome in idiopathic adult motor neuron disease. J Neurol Sci. 1993;118(1):48–55. 8229050 10.1016/0022-510x(93)90245-t

[8] Lopes J, Russell DM, Whitwell J, Jeejeebhoy KN. Skeletal muscle function in malnutrition. Am J Clin Nutr. 1982;36(4):602–10. 6812409 10.1093/ajcn/36.4.602

[9] Rigaud D, Moukaddem M, Cohen B, Malon D, Reveillard V, Mignon M. Refeeding improves muscle performance without normalization of muscle mass and oxygen consumption in anorexia nervosa patients. Am J Clin Nutr. 1997;65(6):1845–51. 9174482 10.1093/ajcn/65.6.1845

[10] Mazzini L, Corra T, Zaccala M, Mora G, Del Piano M, Galante M. Percutaneous endoscopic gastrostomy and enteral nutrition in amyotrophic lateral sclerosis. J Neurol. 1995;242(10):695–8. 8568533 10.1007/BF00866922

[11] Mitsumoto H, Davidson M, Moore D, Gad N, Brandis M, Ringel S, et al Percutaneous endoscopic gastrostomy (PEG) in patients with ALS and bulbar dysfunction. Amyotroph Lateral Scler Other Motor Neuron Disord. 2003;4(3):177–85. 13129795 10.1080/14660820310011728

[12] Russell TR, Brotman M, Norris F. Percutaneous gastrostomy. A new simplified and cost-effective technique. Am J Surg. 1984;148(1):132–7. 6430111 10.1016/0002-9610(84)90300-3

[13] Bradley WG, Anderson F, Bromberg M, Gutmann L, Harati Y, Ross M, et al Current management of ALS: comparison of the ALS CARE Database and the AAN Practice Parameter. The American Academy of Neurology. Neurology. 2001;57(3):500–4. 11502920 10.1212/wnl.57.3.500

[14] Mitchell SL, Tetroe JM. Survival after percutaneous endoscopic gastrostomy placement in older persons. J Gerontol A Biol Sci Med Sci. 2000;55(12):M735–9. 11129395 10.1093/gerona/55.12.m735

[15] Moher D, Liberati A, Tetzlaff J, Altman DG. Preferred reporting items for systematic reviews and meta-analyses: the PRISMA statement. PLoS Med. 2009;6(7):e1000097 doi: 10.1371/journal.pmed.1000097 19621072 10.1371/journal.pmed.1000097 PMC2707599

[16] Wells G, Shea B, O'Connell D, Peterson j, Welch V, Losos M, et al The Newcastle–Ottawa Scale (NOS) for assessing the quality of non-randomized studies in meta-analysis. Ottawa (ON): Ottawa Hospital Research Institute 2009; 2000. Available from: http://www.ohri.ca/programs/clinical_epidemiology/oxford.htm.

[17] DerSimonian R, Laird N. Meta-analysis in clinical trials. Control Clin Trials. 1986;7(3):177–88. 3802833 10.1016/0197-2456(86)90046-2

[18] Ades AE, Lu G, Higgins JP. The interpretation of random-effects meta-analysis in decision models. Med Decis Making. 2005;25(6):646–54. doi: 10.1177/0272989X05282643 16282215 10.1177/0272989X05282643

[19] Deeks JJ, Higgins JPT, Altman DG. Analyzing data and undertaking meta-analyses. In: Higgins J, Green S, editors. Cochrane Handbook for Systematic Reviews of Interventions 501. Oxford, UK: The Cochrane Collaboration; 2008. pp. chap 9.

[20] Higgins JP, Thompson SG, Deeks JJ, Altman DG. Measuring inconsistency in meta-analyses. BMJ. 2003;327(7414):557–60. doi: 10.1136/bmj.327.7414.557 12958120 10.1136/bmj.327.7414.557 PMC192859

[21] Tobias A. Assessing the influence of a single study in meta-analysis. Stata Tech Bull. 1999;47:15–7.

[22] Deeks JJ, Altman DG, Bradburn MJ. Statistical methods for examining heterogeneity and combining results from several studies in meta-analysis In: Egger M, G. DS, Altman DG, editors. Systematic Reviews in Health Care: Metaanalysis in Context. 2nd ed. London: BMJ Books; 2001 pp. 285–312.

[23] Egger M, Davey Smith G, Schneider M, Minder C. Bias in meta-analysis detected by a simple, graphical test. BMJ. 1997;315(7109):629–34. 9310563 10.1136/bmj.315.7109.629 PMC2127453

[24] Begg CB, Mazumdar M. Operating characteristics of a rank correlation test for publication bias. Biometrics. 1994;50(4):1088–101. 7786990

[25] Shaw AS, Ampong MA, Rio A, Al-Chalabi A, Sellars ME, Ellis C, et al Survival of patients with ALS following institution of enteral feeding is related to pre-procedure oximetry: a retrospective review of 98 patients in a single centre. Amyotroph Lateral Scler. 2006;7(1):16–21. doi: 10.1080/14660820510012013 16546754 10.1080/14660820510012013

[26] Mathus-Vliegen LM, Louwerse LS, Merkus MP, Tytgat GN, Vianney de Jong JM. Percutaneous endoscopic gastrostomy in patients with amyotrophic lateral sclerosis and impaired pulmonary function. Gastrointest Endosc. 1994;40(4):463–9. 7926537 10.1016/s0016-5107(94)70211-x

[27] Spataro R, Ficano L, Piccoli F, La Bella V. Percutaneous endoscopic gastrostomy in amyotrophic lateral sclerosis: effect on survival. J Neurol Sci. 2011;304(1–2):44–8. doi: 10.1016/j.jns.2011.02.016 21371720 10.1016/j.jns.2011.02.016

[28] Blondet A, Lebigot J, Nicolas G, Boursier J, Person B, Laccoureye L, et al Radiologic versus endoscopic placement of percutaneous gastrostomy in amyotrophic lateral sclerosis: multivariate analysis of tolerance, efficacy, and survival. J Vasc Interv Radiol. 2010;21(4):527–33. doi: 10.1016/j.jvir.2009.11.022 20172742 10.1016/j.jvir.2009.11.022

[29] Thornton FJ, Fotheringham T, Alexander M, Hardiman O, McGrath FP, Lee MJ. Amyotrophic lateral sclerosis: enteral nutrition provision—endoscopic or radiologic gastrostomy? Radiology. 2002;224(3):713–7. doi: 10.1148/radiol.2243010909 12202704 10.1148/radiol.2243010909

[30] Allen JA, Chen R, Ajroud-Driss S, Sufit RL, Heller S, Siddique T, et al Gastrostomy tube placement by endoscopy versus radiologic methods in patients with ALS: a retrospective study of complications and outcome. Amyotroph Lateral Scler Frontotemporal Degener. 2013;14(4):308–14. doi: 10.3109/21678421.2012.751613 23286755 10.3109/21678421.2012.751613

[31] Desport JC, Mabrouk T, Bouillet P, Perna A, Preux PM, Couratier P. Complications and survival following radiologically and endoscopically-guided gastrostomy in patients with amyotrophic lateral sclerosis. Amyotroph Lateral Scler Other Motor Neuron Disord. 2005;6(2):88–93. doi: 10.1080/14660820410021258 16036431 10.1080/14660820410021258

[32] ProGas Study Group. Gastrostomy in patients with amyotrophic lateral sclerosis (ProGas): a prospective cohort study. Lancet Neurol. 2015;14(7):702–9. doi: 10.1016/S1474-4422(15)00104-0 26027943 10.1016/S1474-4422(15)00104-0 PMC4578147

[33] Chio A, Galletti R, Finocchiaro C, Righi D, Ruffino MA, Calvo A, et al Percutaneous radiological gastrostomy: a safe and effective method of nutritional tube placement in advanced ALS. J Neurol Neurosurg Psychiatry. 2004;75(4):645–7. doi: 10.1136/jnnp.2003.020347 15026518 10.1136/jnnp.2003.020347 PMC1739007

[34] Desport JC, Torny F, Lacoste M, Preux PM, Couratier P. Hypermetabolism in ALS: correlations with clinical and paraclinical parameters. Neurodegener Dis. 2005;2(3–4):202–7. doi: 10.1159/000089626 16909026 10.1159/000089626

[35] Bouteloup C, Desport JC, Clavelou P, Guy N, Derumeaux-Burel H, Ferrier A, et al Hypermetabolism in ALS patients: an early and persistent phenomenon. J Neurol. 2009;256(8):1236–42. doi: 10.1007/s00415-009-5100-z 19306035 10.1007/s00415-009-5100-z

[36] Vaisman N, Lusaus M, Nefussy B, Niv E, Comaneshter D, Hallack R, et al Do patients with amyotrophic lateral sclerosis (ALS) have increased energy needs? J Neurol Sci. 2009;279(1–2):26–9. doi: 10.1016/j.jns.2008.12.027 19185883 10.1016/j.jns.2008.12.027

[37] Genton L, Viatte V, Janssens JP, Heritier AC, Pichard C. Nutritional state, energy intakes and energy expenditure of amyotrophic lateral sclerosis (ALS) patients. Clin Nutr. 2011;30(5):553–9. doi: 10.1016/j.clnu.2011.06.004 21798636 10.1016/j.clnu.2011.06.004

