# Supplementary material for: Therapeutic effects of percutaneous endoscopic gastrostomy on survival in patients with amyotrophic lateral sclerosis: A meta-analysis
Source: PLoS One. 2018 Feb 6;13(2):e0192243. doi: 10.1371/journal.pone.0192243 (PMC5800689; doi:10.1371/journal.pone.0192243)
Supplement: S1 File — (DOC) [file pone.0192243.s002.doc]

(“Percutaneous endoscopic gastrostomy” OR PEG) AND ("Amyotrophic Lateral Sclerosis" [Mesh] OR “sclerosis, amyotrophic lateral” OR “Charcot disease” OR “motor neuron disease, amyotrophic lateral sclerosis” OR “Lou Gehrig disease” OR “disease, Lou Gehrig’s” OR “ALS” OR “amyotrophic lateral sclerosis” OR “Gehrig disease” OR “amyotrophic lateral sclerosis, Guam form” OR “amyotrophic lateral sclerosis, parkinsonism-dementia complex of Guam” OR “Guam form of amyotrophic lateral sclerosis” OR “amyotrophic lateral sclerosis-parkinsonism-dementia complex 1” OR “Guam disease” OR “disease, Guam” OR “amyotrophic lateral sclerosis with dementia” OR “dementia with amyotrophic lateral sclerosis”) OR ("Motor Neuron Disease" [Mesh] OR “motor neuron diseases” OR “neuron disease, motor” OR “motor system disease” OR “familial motor neuron disease” OR “motor neuron disease, familial” OR “lateral sclerosis” OR “scleroses, lateral” OR “primary lateral sclerosis” OR “lateral sclerosis, primary” OR “primary lateral sclerosis” OR “scleroses, primary lateral” OR “motor neuron disease, upper” OR “upper motor neuron disease” OR “motor neuron disease, secondary” OR “secondary motor neuron disease” OR “anterior horn cell disease” OR “motor neuron disease, lower” OR “lower motor neuron disease”)
