# Supplementary material for: Therapeutic effects of percutaneous endoscopic gastrostomy on survival in patients with amyotrophic lateral sclerosis: A meta-analysis
Source: PLoS One. 2018 Feb 6;13(2):e0192243. doi: 10.1371/journal.pone.0192243 (PMC5800689; doi:10.1371/journal.pone.0192243)
Supplement: S1 Table — (DOC) [file pone.0192243.s003.doc]

S1 Table. Meta-regression for 30-day, 10-month, 20-month, and 30-month survival rates according to sample size, mean age, and percentage male.

| Outcomes | Sample size | Mean age | Percentage male |
| --- | --- | --- | --- |
| 30-day survival rate | 0.449 | 0.431 | 0.559 |
| 10-month survival rate | 0.996 | 0.245 | 0.747 |
| 20-month survival rate | 0.806 | 0.959 | 0.540 |
| 30-month survival rate | 0.929 | 0.663 | 0.545 |
